# The Effect of a Single Bout of Exercise on Vitamin B2 Status Is Not Different between High- and Low-Fit Females

**DOI:** 10.3390/nu13114097

**Published:** 2021-11-16

**Authors:** Joëlle J. E. Janssen, Bart Lagerwaard, Arie G. Nieuwenhuizen, Silvie Timmers, Vincent C. J. de Boer, Jaap Keijer

**Affiliations:** 1 Human and Animal Physiology, Wageningen University and Research, P.O. Box 338, 6700 AH Wageningen, The Netherlands; joelle.janssen@wur.nl (J.J.E.J.); bart.lagerwaard@wur.nl (B.L.); arie.nieuwenhuizen@wur.nl (A.G.N.); silvie.timmers@wur.nl (S.T.); vincent.deboer@wur.nl (V.C.J.d.B.); 2 Cell Biology and Immunology, Wageningen University and Research, P.O. Box 338, 6700 AH Wageningen, The Netherlands; 3 TI Food and Nutrition, P.O. Box 557, 6700 AN Wageningen, The Netherlands

**Keywords:** vitamin B2 status, exercise, EGRAC, erythrocyte glutathione reductase, high- and low-fit females

## Abstract

High-fitness individuals have been suggested to be at risk of a poor vitamin B2 (riboflavin) status due to a potentially higher vitamin B2 demand, as measured by the erythrocyte glutathione reductase (EGR) activation coefficient (EGRAC). Longer-term exercise interventions have been shown to result in a lower vitamin B2 status, but studies are contradictory. Short-term exercise effects potentially contribute to discrepancies between studies but have only been tested in limited study populations. This study investigated if vitamin B2 status, measured by EGRAC, is affected by a single exercise bout in females who differ in fitness levels, and that represents long-term physical activity. At baseline and overnight after a 60-min cycling bout at 70% $\overset{\cdot }{V}$O_2_peak, EGR activity and EGRAC were measured in 31 young female adults, divided into a high-fit ($\overset{\cdot }{V}$O_2_peak ≥ 47 mL/kg/min, N = 15) and low-fit ($\overset{\cdot }{V}$O_2_peak ≤ 37 mL/kg/min, N = 16) group. A single exercise bout significantly increased EGR activity in high-fit and low-fit females (P_time_ = 0.006). This response was not affected by fitness level (P_time*group_ = 0.256). The effect of exercise on EGRAC was not significant (P_time_ = 0.079) and not influenced by EGR activity. The exercise response of EGRAC was not significantly different between high-fit and low-fit females (P_time*group_ = 0.141). Thus, a single exercise bout increased EGR activity, but did not affect EGRAC, indicating that vitamin B2 status was not affected. The exercise response on EGRAC and EGR did not differ between high-fit and low-fit females.

## 1. Introduction

Exercise requires chemical energy (adenosine triphosphate, ATP) to enable muscle contractions and relaxations [1]. At the same time, exercise generates reactive oxygen species (ROS) as a by-product, which enhance antioxidant defense systems [2,3,4], including those that depend on glutathione [3]. One essential gatekeeper of energy and redox metabolism is vitamin B2 (riboflavin) [5]. Vitamin B2 acts as a precursor for the electron carriers flavin mononucleotide (FMN) and flavin adenine dinucleotide (FAD), which are both essential mitochondrial cofactors of oxidative phosphorylation (OXPHOS) complexes I and II, respectively [6,7]. In addition, FAD is an essential cofactor for the antioxidant enzyme glutathione reductase (GR) [6,8] that catalyzes the conversion of oxidized glutathione (GSSG) to reduced glutathione (GSH), thereby clearing ROS and supporting redox homeostasis. Since aerobic exercise puts a high demand on processes that are essentially dependent on the vitamin B2-derived cofactors FMN and FAD, especially athletes and recreationally active individuals, i.e., high-fitness (high-fit) individuals, are thought to benefit from an optimal vitamin B2 status [9].

In exercise studies, systemic vitamin B2 status is commonly assessed by the erythrocyte glutathione reductase activation coefficient (EGRAC) biomarker [9,10]. This coefficient represents the ratio between erythrocyte GR (EGR) activity in the absence and presence of its cofactor FAD [11,12]. Higher GR activity in the presence of FAD compared to the activity in its absence reflects the incomplete occupancy of GR by FAD and therefore a lower vitamin B2 status [13]. Using this EGRAC assay, studies have demonstrated that longer-term exercise interventions of three weeks up to three months resulted in a lower vitamin B2 status [14,15,16,17,18,19]. However, not all exercise studies confirm these findings [20,21]; one study failed to show a change in vitamin B2 status [20], whereas another study even showed an improved vitamin B2 status following a longer-term exercise intervention [21]. Large observational studies comparing the vitamin B2 status in athletes and recreationally active individuals also show inconsistent results [22,23,24,25,26,27]. Differences in experimental set-up, including the selection of the study population [14,15,16,17,18,19,20,21,22,23,24,26,27,28] and the assessment of subjects’ fitness levels [24,27] could be of importance. However, the inconsistent findings could also be related to alterations in the EGR enzymatic activity itself. EGR enzymatic activity was shown to increase upon a longer-term exercise intervention [21], but has also been found to alter upon a single bout of exercise [21,28,29,30] and importantly, this has been linked to a lower EGRAC [21,28]. This implies that the long- and short-term effects of exercise on vitamin B2 status parameters could differ, but this has not yet been investigated, nor has the effect of long-term exercise on the EGRAC response to short-term exercise.

This study investigates the effect of a single bout of exercise, i.e., short-term exercise, on EGRAC and EGR activity in high-fit compared to low-fit females, i.e., females that differ in long-term physical activity. We chose to study females, because females have been shown to be at risk for a poor vitamin B2 status [31], especially upon exercise [14,16]. We hypothesize that a single bout of exercise affects these vitamin B2 status parameters, and that the longer-term physiological adaptations to regular aerobic exercise will increase vitamin B2 demand, resulting in a higher EGRAC and lower vitamin B2 status in high-fit females as compared to low-fit females in response to a single bout of exercise. 

## 2. Materials and Methods

### 2.1. Ethics Approval

The protocol for collection and handling of human samples was ethically approved by the medical ethical committee of Wageningen University and Research with reference number NL70136.081.19 and registered in the Dutch Trial Register (NL7891). All procedures performed were in accordance with the ethical standards of the institutional and/or national research committee and with the 1964 Helsinki Declaration. Written informed consent was obtained from all individual subjects included in the study.

### 2.2. Study Subjects

Healthy young females (18–28 y of age, BMI 18.5–25 kg/m^2^) were recruited from the local university and community population. Exclusion criteria were: history of cardiovascular, respiratory, hematological or metabolic disease; use of prescribed chronic medication; anemia (hemoglobin concentration >12 g/dL); blood donation within two months before the start of the study; smoking (>5 cigarettes per week); veganism; recreational drug use or over-the-counter drug use during the study; supplement use (performance enhancers or supplements containing vitamin B2); pregnancy or lactating. Subjects were selected if they had a $\overset{\cdot }{V}$O_2_peak ≥47 mL/kg/min (high-fit group) or ≤37 mL/kg/min (low-fit group). This was determined using a maximal exercise test on a bicycle ergometer (Corival CPET, Lode, The Netherlands), and measured using the screening protocol of Lagerwaard et al. (2019) [32]. The power analysis was based on findings from previous studies that examined the effect of exercise interventions on vitamin B2 status [14,16,17]. This yielded a sample size of N = 14 per group using the G*power software program with 90% power (β), 0.05 level of significance (α), a two-tailed confidence interval, and comparing two dependent (paired) means. Taking a 10% drop-out rate into account, this resulted in a sample size of N = 16 per group. Sixteen high-fit subjects (representing physically active, trained individuals) and sixteen low-fit subjects (representing untrained individuals) were included. The $\overset{\cdot }{V}$O_2_peak data and results of the skeletal muscle mitochondrial capacity of these included subjects have been published previously by our group [33]. A total of 111 maximal exercise tests were conducted to result in the desired sample size of 32 (N = 16 per group), i.e., 79 subjects had a $\overset{\cdot }{V}$O_2_peak ≥37 and ≤47 mL/kg/min. The use of oral contraceptives (OC) was not excluded; only the use of monophasic OC containing low synthetic estradiol and progesterone was allowed and was controlled for (N = 7 in the high-fit and N = 6 in the low-fit group). The 17β-estradiol levels were measured using a chemiluminescent immunoassay on a Lumipulse G1200 analyzer (Fujirebio Incl) at the Erasmus Medical Centre (NL) and were not significantly different between those high-fit and low-fit females that did not use OC (Table 1) and were not correlated with basal EGRAC (Supplementary Figure S1).

### 2.3. Study Design

Subjects refrained from heavy physical exercise 48 h prior to the first study day and from any physical exercise and consumption of alcohol 24 h prior to the first study day. Subjects adhered to dietary guidelines 24 h before each study day, which included a list of product choices containing low levels of vitamin B2, and recorded their food intake in a food diary. They also consumed a standardized evening meal (73% carbohydrates/16% protein/11% fat, 1818 kJ) before 8:00 p.m.; subjects were not allowed to eat after 8:00 p.m. After the overnight fast, blood was collected on the morning of the first study day (baseline) and overnight after a single bout of exercise, i.e., the morning of the second study day (21 h post-exercise). Subjects received breakfast and after two hours, subjects completed an individualized exercise protocol consisting of 60-min cycling on an electrically braked bicycle ergometer (Corival CPET, Lode, The Netherlands) at a workload aiming to equal 70% of their individual $\overset{\cdot }{V}$O_2_peak that was determined during the maximal exercise test. Oxygen consumption, carbon dioxide production, and air flow were measured using the MAX-II metabolic cart (AEI technologies, Landivisiau, France). Exhaled air was continuously sampled from a mixing chamber and averaged over 15 s time windows. Oxygen consumption was measured the first and last 15 min of the exercise test and used to confirm the relative workload. Body fat percentage was measured according to the four-site method by Durnin–Womersley using the skinfold measurements of the triceps, biceps, sub scapula, and supra iliac, measured using a skinfold calliper (Harpenden, UK). After the exercise, the protocol subjects went home and refrained from moderate to heavy physical activity, kept low levels of light physical activity, and refrained from alcohol consumption until blood collection on the second study day. Physical activity was monitored using a wearable accelerometer (wGT3X-BT, Actigraph, FL, USA) during this period. Habitual vitamin B2 intake was determined via a food frequency questionnaire (FFQ) that assessed dietary intake in the past four weeks [34]. 

### 2.4. Blood Sampling and Hemolysate Preparation

Blood samples (1 × 6 mL) were collected from the subjects by venapuncture in vacutainers containing dipotassium (K2-) ethylenediaminetetraacetic acid (EDTA) (K2-EDTA, BD Biosciences, Vianen, Netherlands), kept on ice-water, and processed within 30 min. Blood was centrifugated for 10 min at 1200× *g* at 4 °C. Plasma and buffy coat were removed and 2 mL of erythrocytes were collected and transferred to a new sample tube. Erythrocytes were washed twice in 10 mL of 1× Dulbecco’s phosphate-buffered saline (DPBS, Thermo Fisher Scientific, Pittsburgh, PA, USA) and centrifugated for 5 min at 2000 *g* at 4 °C. The supernatant was discarded, and the erythrocyte pellet was gently resuspended in sterile Milli-Q (MQ) to induce an osmotic burst. Hemolysates were stored at −20 °C for 30 min and transferred to −80 °C afterwards. EGRAC assays were performed within six months after blood collection. For the EGRAC assay, hemolysates were thawed on ice, 10× diluted with MQ, and centrifugated for 2 min at 13,000× *g* at 4 °C to remove cellular debris. The supernatant was transferred to a new vial and protein content was determined. Thawed hemolysates and samples were kept on ice and protected from light.

### 2.5. Protein Content Determination

The hemolysate protein content was determined in 100× diluted samples using the DC Protein Assay kit (Bio-Rad, Hercules, CA, USA) according to the manufacturer’s protocol and a standard curve of bovine serum albumin (BSA, Sigma-Aldrich, St. Louis, MO, USA) in Milli-Q. Absorbance was measured using a BioTek Synergy HT plate reader. Hemolysate protein content was optimized (0–300 µg) to achieve linearity of the EGR enzymatic reaction within the 30 min measurement period and the absorbance detection limit. The final protein input was set to 112.5 µg.

### 2.6. Erythrocyte Glutathione Reductase Activity Coefficient (EGRAC) Assay

The EGRAC assay quantifies the reduction of oxidized glutathione (GSSG) to reduced glutathione (GSH) with the concomitant oxidation of NADPH to NADP^+^ in the presence and absence of its essential cofactor FAD [11,12]. The assay was based on the method of Powers et al. (2003) [35] and optimized for the in-house analysis. All reagents were prepared fresh daily. Samples were diluted to 5 µg/µL protein and a 270 µL sample was incubated for 30 min at 37 °C in the presence of 30 µL 15 µM FAD (final concentration 1.5 µM, Sigma-Aldrich, St. Louis, MO, USA) dissolved in 100 mM/3.4 mM H_2_KPO_4_/EDTA buffer pH 7.6 (for FAD-stimulated EGR activity) or with 30 µL H_2_KPO_4_/EDTA buffer only (for unstimulated EGR activity). After incubation, a 25 µL sample was aliquoted in octuplicate in a 96-well microplate (final protein input 112.5 µg). To each sample, 125 µL of the cofactor 0.33 mM beta-nicotinamide adenine dinucleotide (NADPH, final concentration 0.16 mM, Sigma-Aldrich) in H_2_KPO_4_/EDTA buffer (NADPH mix, 37 °C) and 50 µL MQ (37 °C) was added. Absorbance was measured every minute for 5 min at 340 nm at 37 °C using a BioTek Synergy HT plate reader. The enzymatic reaction was started by adding 50 µL 5 mM oxidized glutathione (GSSG, final concentration 1 mM, Sigma-Aldrich, 37 °C) as a substrate. The reaction was monitored every 30 s for 30 min at 340 nm at 37 °C. Reaction slopes (0–30 min) were calculated using a linear regression analysis. Each assay included a reference blood sample to assess intra-assay variation, a positive control (GR from baker’s yeast (6.25 units/µg protein, Sigma-Aldrich) in 1% BSA in H_2_KPO_4_/EDTA buffer), and negative controls for the background, substrate, cofactor, and enzyme. EGR enzymatic activity was calculated using the Beer–Lambert law:

$$C=\frac{\Delta A}{\varepsilon \times \lambda }\;$$

where *c* is the concentration of NADPH (mM), ΔA is the decrease in NADPH absorbance per minute, ε  is the molecular absorbance of NADPH at 340 nm (6.22 × 10^3^ mmol/L/cm), and λ  is the optical pathlength (0.69 cm). Activity was expressed as nmol NADPH oxidized per minute after adjustment for well volume (250 µL). The EGRAC ratio was calculated as FAD-stimulated EGR activity/unstimulated EGR activity using the reaction slopes (all R^2^ > 0.99). An EGRAC of 1.0 reflected complete FAD saturation and EGRAC > 1.3 was used as a cut-off for suboptimal vitamin B2 status [10]. The intra-assay coefficient of variation (CV) for EGR activity was 3.7%; the inter-assay CV was 2.8% for EGRAC and 12.3% for EGR activity.

### 2.7. Statistical Analyses

Statistical analyses were performed using IBM SPSS Statistics for Windows (Version 25.0, IBM Corp, Armonk, NY, USA). Graphs were created using GraphPad Prism (Version 8.0, Graphpad Software, CA, USA). Data were presented as mean ± standard deviation (SD) or as median ± interquartile range (IQR). Normality was checked using Shapiro–Wilk normality tests. A repeated-measures ANOVA (RM-ANOVA) was used to study the effect of a single exercise bout on the response of EGRAC and EGRAC-related parameters, with the exercise bout as the within-subjects factor (time) and fitness level as the between-subjects factor (group). All assumptions for the RM-ANOVA analysis were met. Pearson correlation coefficients (r) were used to compare associations between variables, and *p*-values < 0.05 were considered as statistically significant. 

## 3. Results

In total, 31 of the 32 subjects finished the study protocol. One subject was excluded due to protocol violation (i.e., medication intake). The conducted $\overset{\cdot }{V}$O_2_peak measurements were considered valid on the posed criteria. Subject characteristics are shown in Table 1. All submaximal exercise tests were performed at a workload corresponding to approximately 70% $\overset{\cdot }{V}$O_2_peak (67.2 ± 8.1% in high-fit and 71.8 ± 5.7% in low-fit females). Average group values from the exercise test are shown in Supplementary Figure S2. Two subjects from the low-fit group terminated the submaximal exercise test prematurely due to feelings of presyncope and exhaustion; one subject cycled for 40 min at an intensity of 71% and one subject cycled for 48 min and at an intensity of 71.9%. Average habitual vitamin B2 intake was not significantly different between high-fit (1.40 ± 0.35 mg/d) and low-fit (1.32 ± 0.46 mg/d) females (*p* = 0.595).

### 3.1. EGR Enzymatic Activities Were Increased after a Single Bout of Exercise, but Were Not Significantly Different in High-Fit Compared to Low-Fit Females

**Figure 1:**
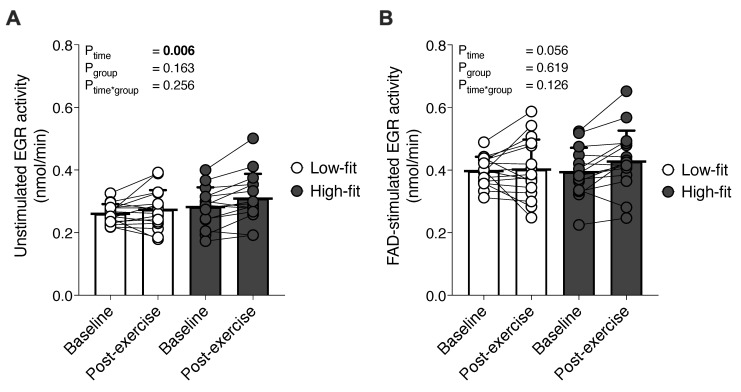
The effect of a single exercise bout on unstimulated and FAD-stimulated EGR activity in high-fit and low-fit females. Baseline and post-exercise unstimulated EGR activity (expressed as nmol NADPH converted per minute (**A**)), and FAD-stimulated EGR activity (expressed as nmol NADPH converted per minute (**B**)), in low-fit (N = 16, white) and high-fit (N = 15, grey) females.

Compared to baseline, a single bout of exercise significantly increased unstimulated EGR activity (P_time_ = 0.006), but fitness level did not influence this outcome (P_group_ = 0.163) (Figure 1A). The exercise-induced increase (0.260 ± 0.031 to 0.273 ± 0.060 nmol NADPH per minute) in unstimulated EGR activity in low-fit females was not significantly different from the increase (0.282 ± 0.063 to 0.309 ± 0.079 nmol NADPH per minute) in high-fit females (P_time*group_ = 0.256). FAD-stimulated EGR activity increased after the exercise bout, but this did not reach statistical significance (P_time_ = 0.056) (Figure 1B). Again here, fitness level had no influence on this finding (P_group_ = 0.619). The exercise-induced increase in FAD-stimulated EGR activity in low-fit females (0.397 ± 0.046 to 0.401 ± 0.096 nmol NADPH per minute) was not significantly different from the increase (0.393 ± 0.078 to 0.427 ± 0.100 nmol NADPH per minute) in high-fit females (P_time*group_ = 0.126). 

### 3.2. The EGRAC Response to a Single Bout of Exercise Is Not Different between High-Fit and Low-Fit Females and Not Related to the EGR Response

**Figure 2:**
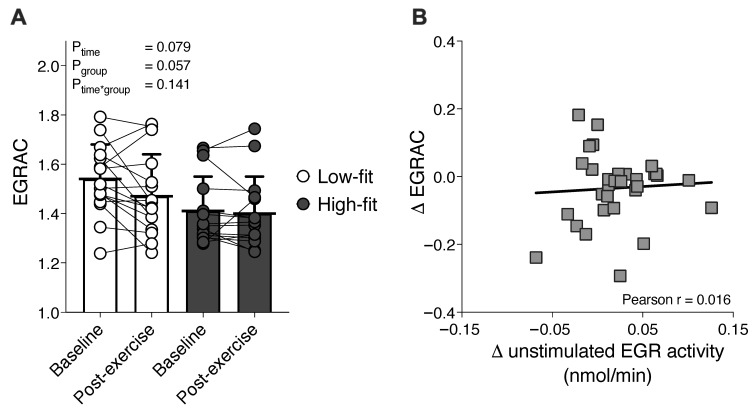
The effect of a single exercise bout on vitamin B2 status (EGRAC) in high-fit and low-fit females. Baseline and post-exercise EGRAC in low-fit (N = 16, white) and high-fit (N = 15, grey) females (**A**). Correlation between the individual exercise response of unstimulated EGR activity (Δ unstimulated EGR activity, expressed as nmol NADPH per minute) and the exercise response of EGRAC (Δ EGRAC); the high-fit and low-fit groups are plotted together (**B**).

A single bout of exercise did not affect the vitamin B2 status as reflected by EGRAC (P_time_ = 0.079), and fitness level did not have a significant effect (P_group_ = 0.057) (Figure 2A). The exercise-induced decrease (1.54 ± 0.14 to 1.47 ± 0.17) in EGRAC in low-fit females was not significantly different from the decrease (1.41 ± 0.14 to 1.40 ± 0.15) in high-fit females (P_time*group_ = 0.141). The individual exercise responses of EGRAC and unstimulated EGR activity were analyzed per subject and plotted against each other with the two groups combined. The change in unstimulated EGR activity after exercise (Δ unstimulated EGR activity) was not correlated (r = 0.06, P = 0.74) to the change in EGRAC after exercise (Δ EGRAC) (Figure 2B).

## 4. Discussion

Our results indicate that the effect of a single bout of exercise on EGRAC and EGR was not different between high- and low-fit females. The exercise bout significantly increased unstimulated EGR activity and tended to increase FAD-stimulated EGR activity, yet this exercise response was not different between high-fit and low-fit females. The decrease in EGRAC in response to a bout of exercise was not significant; neither was this response significantly different between high-fit and low-fit females. There was a lack of association between the change in EGR and EGRAC in response to a bout of exercise. 

We hypothesized that the single bout of exercise could affect vitamin B2 status as determined by EGRAC, as the short-term effects (<24 h) of exercise on EGR activity have been reported by previous studies [21,28,30]. We also hypothesized that the EGRAC values after the single bout of exercise would be higher, i.e., vitamin B2 status would be lower in high-fit compared to low-fit individuals, as regular aerobic exercise enhances vitamin B2-dependent processes that could increase vitamin B2 demand. Indeed, we observed that EGR activity was significantly increased after the single bout of exercise, but EGRAC was not significantly affected, neither was the change in unstimulated EGR activity related to the change in EGRAC. The first finding implies that a short-term exercise intervention on the day prior to blood sampling does not affect the reliability of using EGRAC for vitamin B2 status determination. Moreover, our observations are similar to previous findings [21,28,30]. Ohno et al. [30] demonstrated in 11 untrained males (age 20.3 ± 0.3 y) that a single bout of exercise (30-min cycling test at 75% $\overset{\cdot }{V}$O_2_peak) significantly increased FAD-stimulated EGR activity and non-significantly increased unstimulated EGR activity [30], but EGRAC values were lacking. Evelo et al. [21] observed significantly higher EGR activity and significantly decreased EGRAC in trained males and females (N = 23 and 18, age 18–41 y) one day after running a 15-km contest or a half marathon. Similar effects of exercise on EGR activity and EGRAC were found by Frank et al. [28] in females (N = 5) and males (N = 5 and 55, age 25–65 y) that participated in a 100-km walking contest and showed a non-significantly increased EGR activity and a significantly decreased EGRAC. These two studies cited above [21,28] used longer-duration exercises, i.e., a 15-km running contest or 100-km walking contest, which may explain why they found a significant effect on EGRAC, while our results were not significant. Despite this, previous studies have thus also consistently shown that short-term exercise elevated EGR activity [21,28,30] and decreased EGRAC [21,28], yet we are the first study that examined this effect in both high-fit and low-fit individuals. High-fit and low-fit individuals could have responded differently to the single bout of exercise, as the redox metabolism can adapt in response to regular aerobic training [2,36]. That is, the increase in exercise-induced oxidative damage seems higher in untrained compared to trained individuals [36]. Consequently, a single bout of exercise could have resulted in different redox enzyme activity between high-fit and low-fit females; however, our study indicated that the response of EGR activity and EGRAC to a single bout of exercise did not significantly differ between the two groups. Furthermore, our study demonstrates that the change in EGR activity was not correlated to the change in EGRAC after exercise. Apparently, short-term exercise could influence EGR activity without affecting EGR saturation with FAD in the short-term. Fitness level does not seem to be important, indicating that other factors, such as vitamin B2 intake [28,37,38], could be more important determining factors for short-term EGRAC responses. In our study, the habitual vitamin B2 intake was not different between high-fit and low-fit females; therefore, this may have resulted in similar EGRAC responses between the two groups.

Our results indicated that fitness level did not significantly affect EGRAC. Studies have described that vitamin B2-dependent processes can adapt in response to training, including an increased mitochondrial number and function [39,40] and an increased production of FAD- and FMN-dependent enzymes, such as succinate dehydrogenase and long-chain acyl-CoA dehydrogenase, but also GR [41,42,43], which could increase vitamin B2 flux. Several longer-term exercise intervention studies have translated this into an increased vitamin B2 requirement for athletes and physically active individuals in response to regular aerobic exercise [14,15,16,17,18]. This was supported by increased EGRAC after regular aerobic exercise while vitamin B2 intake was controlled [14,15,16,17,18], but was not confirmed by other intervention studies [20,21,28]. Observational studies also did not find EGRAC differences between trained and untrained subjects [23,24,25], which is similar to the lack of a fitness level effect in our study. Based on the results of our study, we suggest that regular aerobic training could possibly change vitamin B2 status, but that the training adaptations in high-fit individuals do not necessarily result in an increased vitamin B2 requirement during exercise; if this was the case, we would likely have found a difference in the EGRAC response to the single exercise bout between high-fit and low-fit females, as well as increased rather than decreased EGRAC after exercise. We cannot exclude that EGRAC may change following more strenuous exercise performed over a longer period of time, since EGRAC was found to be significantly decreased following a 15-km running contest or 100-km walking contest [21,28]. However, in those cases a decrease rather than an increase in EGRAC was observed. 

Our findings suggest that vitamin B2 status was suboptimal (EGRAC > 1.3) in almost all females (15 out of 16 low-fit and 13 out of 15 high-fit females). The risks of vitamin B2 deficiency have been observed in other exercise studies [14,15,16,18,19,20,44,45], but also in other study populations, including adolescents, young women, adults, and the elderly from developed countries such as the United Kingdom, Spain, and France [31,46,47,48,49]. In The Netherlands, vitamin B2 status studies are scarce; one cross-sectional study from 1989 showed that the mean EGRAC in adult (18–64 y) females was 1.11 [50], and the first Dutch food consumption survey from 1987–1988 showed that the average vitamin B2 intake among females aged 22–49 y was 1.49 mg/d [51]. The mean habitual vitamin B2 intake across subjects in our study was close to the RDA of 1.4 mg [10,52], and it is thus remarkable that we found a suboptimal vitamin B2 status. The controlled, low vitamin B2 intake during the study could possibly play a role, although we deem this unlikely since EGRAC is considered a vitamin B2 status rather than a vitamin B2 intake marker [10]. Furthermore, an EGRAC > 1.3 is generally accepted as a cut-off point for a suboptimal vitamin B2 status [10], but cut-off points of 1.2 to 1.4 have also been used [13,46,48]. Details in experimental protocols between studies differ [11,12,53], which are unlikely to affect relative EGRAC outcomes, but may impact absolute EGRAC values. This has been proposed earlier by Hill et al. [35], who indicated that details of the EGRAC method should be standardized and the EGRAC cut-off point should be re-evaluated. Although this was not the focus of our study, we recommend future studies to focus on validation of the EGRAC cut-off point to better understand the consequences of a suboptimal vitamin B2 status, especially since it may dispose a person to enhanced (metabolic) disease susceptibility. 

The strength of our study is the use of a well-characterized study population including high-fit and low-fit individuals with a validated difference in $\overset{\cdot }{V}$O_2_peak, while previous studies included only a high-fit or low-fit group that received exercise interventions [14,15,16,17,21,28,30] or selected their study population by exercise routines or physical activity questionnaires [20,24,27,44]. Another strength of our study is that we sampled blood the day after the exercise intervention to mimic a situation that allowed us to examine if the recent effects of exercise could interfere with the EGRAC determination on the next day. A limitation is that we only sampled at one timepoint, while more insight may have been obtained by a time-course since the effects of exercise on EGR can be maintained up to 24 h after exercise [21] and the kinetics may differ between individuals. Another limitation is the assessment of habitual vitamin B2 intake using the FFQ, which is based on selected foods from the food consumption data of the Dutch National Food Consumption Survey of 1998 [34], but may be influenced by the inter-individual variation in the relatively small population used in our study. Lastly, our data did not show a significant effect of fitness level on EGRAC. The relatively small sample size of our study may have contributed to this. Vitamin B2 status tended to be better rather than worse in high-fit females, which would possibly have statistically been confirmed with a larger sample size.

In conclusion, the effect of a single bout of exercise on EGRAC and EGR was not different between high-fit and low-fit females. A single bout of exercise significantly increased EGR activity, but did not affect EGRAC values, indicating that a single bout of exercise did not affect vitamin B2 status. Our findings help to better understand the influence of short-term exercise on vitamin B2 status and contribute to the interpretation of EGRAC as a vitamin B2 status parameter.

## Figures and Tables

**Table 1:** Subject characteristics.

	Low-Fit (N = 16)	High-Fit (N = 15)
Age (y)	24.0 (21.3–25.5)	21.8 (21.6–23.7)
Weight (kg)	59.2 ± 7.2	61.2 ± 7.0
Height (m)	1.63 ± 0.08	1.68 ± 0.05 *
Fat mass (% of weight)	28.9 ± 3.9	25.1 ± 4.4 *
$\overset{\cdot }{V}$O_2_peak (mL/kg/min)	35.1 (32.2–35.7)	50.4 (49.0–54.0) ****
Baecke total score	7.3 ± 1.0	9.5 ± 0.8 ****
Hemoglobin (mmol/L)	8.4 ± 0.6	8.5 ± 0.6
Use of birth control pill	6/16	7/15
If not; 17β-estradiol (pmol/L)	470.9 (337.2–590.1)	217.4 (109.1–895.2)

$\overset{\cdot }{V}$O_2_peak = maximal oxygen consumption values. Values are mean ± SD for normally distributed data, and median [IQR] for not normally distributed data. * *p* < 0.05, **** *p* < 0.0001.

## Data Availability

The data presented in this study are available in this article and its Supplementary Materials.

## Supplementary Materials

The following are available online at https://www.mdpi.com/article/10.3390/nu13114097/s1, Figure S1: The effect of oral contraceptives (OC) use and 17β-estradiol levels on vitamin B2 status, Figure S2: Submaximal exercise test characteristics.
