# Supplementary material for: The Effect of a Single Bout of Exercise on Vitamin B2 Status Is Not Different between High- and Low-Fit Females
Source: Nutrients. 2021 Nov 16;13(11):4097. doi: 10.3390/nu13114097 (PMC8618623; doi:10.3390/nu13114097)
Supplement: Supplementary file 1 [file nutrients-13-04097-s001.zip › nutrients-1422035 supplementary.pdf]

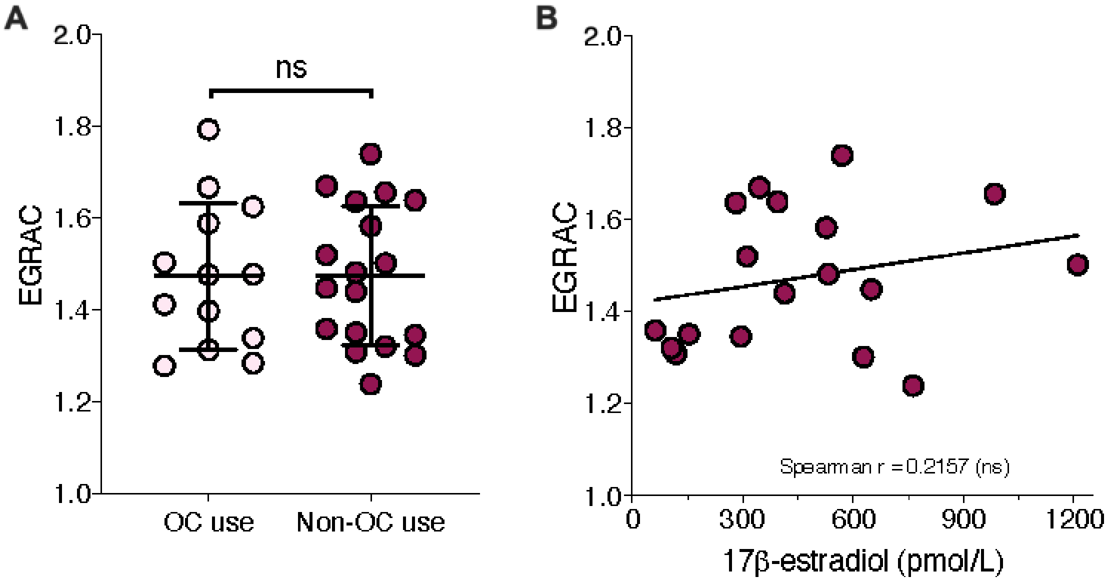

Supplementary Fig S1: The effect of oral contraceptives (OC) use and 17β-estradiol levels on vitamin B2 status. (A) Baseline EGRAC in OC users (N = 13, light pink) and non- OC users (N = 18, dark pink). (B) Correlation between levels of 17β-estradiol and baseline EGRAC in OC users (N = 18). ns = nonsignificant

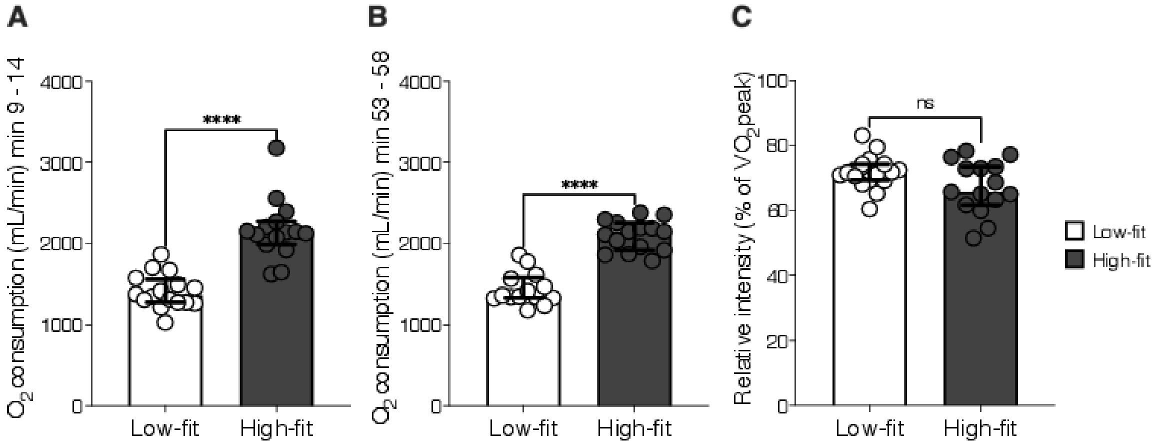

Supplementary Fig S2: Submaximal exercise test characteristics The average oxygen consumption (mL/min) was calculated between 9-14 minutes in low-fit (N = 16, white) and high- fit (N = 15, grey) females (A) and between 5358 minutes in low-fit (N = 14, whitr) and low-fit (N = 15, grey) females of the 60 minutes exercise protocol(B). (C) The mean intensity of the exercise test as a percentage to an individual's VO_2 peak. ns = nonsignificant. **** $p < 0.001$
